# Functional Assay of Cancer Cell Invasion Potential Based on Mechanotransduction of Focused Ultrasound

**DOI:** 10.3389/fonc.2017.00161

**Published:** 2017-08-07

**Authors:** Andrew C. Weitz, Nan Sook Lee, Chi Woo Yoon, Adrineh Bonyad, Kyo Suk Goo, Seaok Kim, Sunho Moon, Hayong Jung, Qifa Zhou, Robert H. Chow, K. Kirk Shung

**Affiliations:** ^1^Ultrasonic Transducer Resource Center, University of Southern California, Los Angeles, CA, United States; ^2^Institute for Biomedical Therapeutics, University of Southern California, Los Angeles, CA, United States; ^3^Zilkha Neurogenetic Institute, University of Southern California, Los Angeles, CA, United States; ^4^USC Roski Eye Institute, University of Southern California, Los Angeles, CA, United States

**Keywords:** cancer invasion, focused ultrasound, calcium imaging, bladder cancer, prostate cancer

## Abstract

Cancer cells undergo a number of biophysical changes as they transform from an indolent to an aggressive state. These changes, which include altered mechanical and electrical properties, can reveal important diagnostic information about disease status. Here, we introduce a high-throughput, functional technique for assessing cancer cell invasion potential, which works by probing for the mechanically excitable phenotype exhibited by invasive cancer cells. Cells are labeled with fluorescent calcium dye and imaged during stimulation with low-intensity focused ultrasound, a non-contact mechanical stimulus. We show that cells located at the focus of the stimulus exhibit calcium elevation for invasive prostate (PC-3 and DU-145) and bladder (T24/83) cancer cell lines, but not for non-invasive cell lines (BPH-1, PNT1A, and RT112/84). In invasive cells, ultrasound stimulation initiates a calcium wave that propagates from the cells at the transducer focus to other cells, over distances greater than 1 mm. We demonstrate that this wave is mediated by extracellular signaling molecules and can be abolished through inhibition of transient receptor potential channels and inositol trisphosphate receptors, implicating these proteins in the mechanotransduction process. If validated clinically, our technology could provide a means to assess tumor invasion potential in cytology specimens, which is not currently possible. It may therefore have applications in diseases such as bladder cancer, where cytologic diagnosis of tumor invasion could improve clinical decision-making.

## Introduction

Cancer staging determines both patient prognosis and treatment protocol. To stage a biopsied tumor, the pathologist must determine the extent to which the tumor has invaded the surrounding tissue. In many instances, however, the intact tissue needed to assess invasion cannot be obtained from the patient. In such cases, fine-needle aspirations, washings, or brushings can be performed to collect cells from the tumor for cytologic diagnosis. These cells allow the cytopathologist to determine whether the tumor is benign or malignant, but not whether it is invasive.

The inability to assess invasion can have devastating consequences, for example in the case of bladder cancer. Bladder tumors that have begun to invade the muscle wall must be treated promptly with cystectomy (surgical removal of the bladder), or they may become life-threatening (1). Carcinoma *in situ* (CIS) is an early form of bladder cancer that is considered high grade, as these tumors frequently recur as muscle-invasive disease (2). CIS is normally treated with bacillus Calmette–Guérin (BCG) immunotherapy upon initial diagnosis and recurrence (3). However, BCG inflames the bladder epithelium, making it difficult endoscopically to identify recurrent tumors for biopsy (4). In such cases, bladder wash cytology can be used to detect recurrence, but the invasion status of the recurrent malignancy cannot be determined. The inability to detect invasion precludes the use of preventative cystectomy, which can have fatal consequences if the cancer is indeed invasive. Thus, a method for assessing tumor invasion cytologically (e.g., in bladder washings) would enable appropriate and timely treatments that improve patient outcomes.

Classical cytology relies on examining cell morphology to identify the presence and appearance of malignant cells. Biophysical properties of tumor cells could reveal information about their malignant status that might escape detection in morphological studies. Recent work has revealed a number of biophysical changes that occur during cancer transformation and progression (5). For example, metastatic cells often express voltage-gated ion channels, including the Na^+^ (6), K^+^ (7), and Ca^2+^ (8) types, rendering them electrically excitable. Similarly, mechanical properties of metastatic cancer cells differ from those of benign cells; metastatic cells are generally “softer” (9). Because these biophysical changes can serve as diagnostic markers, assays to measure biophysical properties of tumor cells have been proposed and are under development (9–11). These assays are typically intended to differentiate malignant from non-malignant cells in suspension, thus providing information similar to that obtained *via* standard cytological analysis.

Here, we present a new biophysical cancer assay that, to our knowledge, is the first that can assess the invasion potential of isolated tumor cells. The assay leverages the fact that cancer progression is accompanied by remodeling of calcium channels (8, 12, 13) and cellular mechanosensors (14). The assay applies a non-contact, mechanical stimulus—low-intensity focused ultrasound—to probe for presence of these proteins while monitoring their activity *via* calcium imaging. The stimulus elicits marked calcium elevations in invasive cancer cells, but not in non-invasive cells. We previously validated this assay in four breast cancer cell lines and demonstrated its effectiveness in quantifying invasion potential (15). However, analysis was limited to single cells, making the assay impractical for a clinical setting.

In the present study, we show that our assay can be used for high-throughput, functional analysis of cancer invasion in cell populations. A single ultrasound stimulus is applied while monitoring calcium activity in hundreds or thousands of cells simultaneously. We validate the technique in prostate and bladder cancer cell lines while exploring the role of different stimulus parameters. We also investigate the mechanism by which ultrasound elicits calcium elevations in invasive cells. If validated through further testing, this technology may lend itself to cytological assessment of tumor invasion, thus having important implications for diagnosis and management of diseases such as bladder cancer.

## Materials and Methods

### Cell Lines

Six human cancer cell lines were used in this study: four prostate cancer lines (PC-3, DU-145, BPH-1, and PNT1A) and two bladder cancer lines (T24/83 and RT112/84). PC-3 cells were obtained from Frank Markland (University of Southern California), DU-145 and PNT1A from Mitchell Gross (University of Southern California), BPH-1 from Simon Hayward (NorthShore University HealthSystem), and T24/83 and RT112/84 from Sigma. PC-3 and DU-145 cells were cultured in DMEM, PNT1A, and BPH-1 cells in RPMI-1640, T24/83 cells in McCoy’s 5A medium, and RT112/84 cells in EMEM. All medium was supplemented with 10% FBS and 2 mM l-glutamine, and RT112/84 medium was additionally supplemented with 1% non-essential amino acids. All cell lines were tested to be free of mycoplasma contamination using a mycoplasma PCR detection kit (Sigma). Cell authentication of all lines was performed with Promega’s PowerPlex 16 System within 6 months of use.

PC-3, DU-145, and T24/83 are highly invasive cell lines, while BPH-1, PNT1A, and RT112/84 are weakly invasive (16–19). We confirmed the invasion status of each cell line with Matrigel Boyden chamber assays (20). Invasion potential was measured in BioCoat Matrigel Invasion Chambers (Corning), as described in our previous study (15). In brief, cells (1–1.5 × 10^5^) were added to chambers and incubated for 1–2 days at 37°C. Non-invasive cells at the top of the chamber were removed by a cotton swab, and invasive cells that had passed through the Matrigel were stained with 0.2% crystal violet in 10% ethanol. Three independent fields of invasive cells per well were photographed under a microscope.

Preliminary results showed that BPH-1 cells exhibited variable levels of invasiveness over time, as measured by the Matrigel assay (Figure S1 in Supplementary Material) and our ultrasound assay (data not shown). In order to obtain a weakly invasive, homogeneous cell population, BPH-1 cells that passed through the Boyden chamber were removed after 24 h, and cells that did not pass through were selected and propagated. All BPH-1 experiments were performed with the selected cells, which were confirmed to be weakly invasive (see Figures 2 and 3). Experiments in the other five cell lines did not require selection, as these lines exhibited consistent levels of invasiveness over time.

### Cell Preparation

Cells were plated on 35-mm polystyrene culture dishes to a density of 10^6^ cells per dish. In one experiment, the substrate was coated with Cell-Tak Cell and Tissue Adhesive (Corning) to facilitate immediate cell adhesion. In another experiment, the culture dish substrate was replaced with an acoustically transparent, 50-μm-thick Mylar film (#48-2F-36; CS Hyde Company) to minimize reflection at the surface and eliminate ultrasonic surface waves. All cells were stained with cell-membrane permeant Fluo-4 AM (Thermo Fisher Scientific), a fluorescent reporter of intracellular calcium activity. Staining was performed by incubating dishes with 1 µM Fluo-4 AM for 30–60 min immediately prior to imaging. Following calcium dye loading, cells were washed with and maintained in external buffer solution consisting of 140 mM NaCl, 2.8 mM KCl, 1 mM MgCl_2_, 2 mM CaCl_2_, 10 mM HEPES, and 10 mM d-glucose, adjusted to pH 7.3 and 300 mOsm.

### Ultrasound Transducers

**Figure 1:**
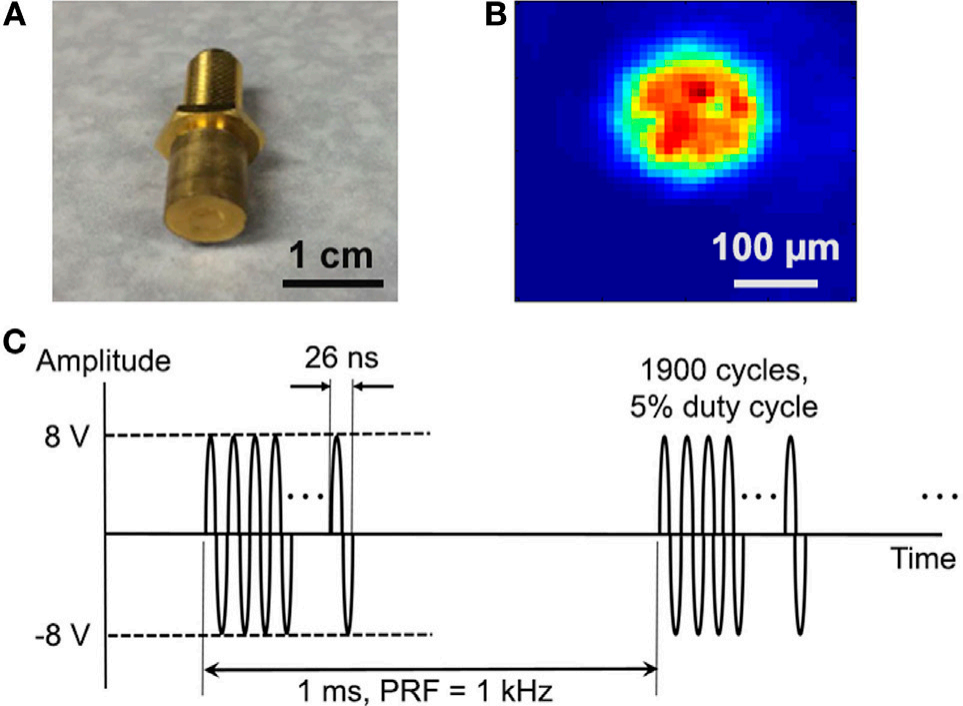
Cancer cells were stimulated with single-element, LiNbO_3_, press-focused transducers. **(A)** Photograph of the 38-MHz transducer used in most experiments. **(B)** Two-dimensional beam profile of *I*_spta_ at the ultrasound focus, as measured by a hydrophone. **(C)** Typical voltage waveform used to drive the transducer. Carrier frequency is 38 MHz, amplitude is 16 V_p–p_, pulse repetition frequency is 1 kHz, and duty cycle is 5%.

Single-element, lithium niobate (LiNbO_3_), press-focused transducers were fabricated in house as described previously (21). A transducer with a center frequency of 38 MHz (f-number = 2, focal length = 8 mm) was used in most experiments (Figure 1A); a 3-MHz transducer (f-number = 2, focal length = 6 mm) was also tested. To drive the transducers, sinusoidal bursts from a signal generator (SG382; Stanford Research Systems) were fed to a 50-dB power amplifier (525LA; Electronics & Innovation) whose output was used to excite the transducer. Unless specified otherwise, amplitude was fixed at 16 V_p–p_, pulse repetition frequency (PRF) at 1 kHz, and duty cycle at 5% (Figure 1C).

The acoustic output of the 38-MHz transducer was measured with a needle hydrophone (HGL-0085; Onda). Figure 1B shows the two-dimensional intensity beam profile, indicating that the diameter of the ultrasound focus is approximately 150 µm. Using the standard cell stimulation parameters provided above (16 V_p–p_ amplitude, 1 kHz PRF, and 5% duty cycle), the intensity and pressure at the focus were measured by the hydrophone to be 353 mW/cm^2^ spatial-peak temporal-average intensity (*I*_spta_), 7.0 W/cm^2^ spatial-peak pulse-average intensity (*I*_sppa_), and 394 kPa peak pressure. The mechanical index was measured to be 0.02. These values are below the FDA safety limit for diagnostic ultrasound (22).

### Ultrasound Stimulation and Fluorescence Imaging

A custom microscope system was used to image cellular fluorescence while performing simultaneous ultrasonic stimulation (15). Petri dishes containing cells were placed on an inverted epifluorescence microscope (Olympus IX70), and the ultrasound transducer was lowered into the external buffer solution. A motorized three-axis micromanipulator was used to position the transducer in focus with the cell monolayer.

In each experiment, live-cell fluorescence imaging was performed for 300 s, with the ultrasound stimulus being delivered continuously between *t* = 50 and 200 s. Excitation light was provided by a mercury arc lamp and filtered through an excitation bandpass filter (488 ± 20 nm). Fluorescence emitted from the calcium dye was filtered through an emission bandpass filter (530 ± 20 nm) and recorded at 1 Hz (30% exposure duty cycle) with a digital CMOS camera (ORCA-Flash2.8; Hamamatsu). All imaging was performed at 4× magnification in order to capture activity from hundreds or thousands of cells simultaneously. For each cell line, simulation and imaging experiments were replicated in at least two different dishes of cells, and over least three independent fields of view per dish. (Experiments involving pharmacological blockers were limited to a single field of view per dish.) Figures show representative data obtained from one field of view.

### Data Processing

Data were post-processed to determine the calcium response of every imaged cell. Cell locations were identified automatically with CellProfiler image analysis software (23) and used to extract the raw fluorescence intensities of each cell. These intensities were exported to MATLAB (MathWorks) in order to calculate each cell’s normalized change in fluorescence (Δ*F*/*F*) during every imaging frame. Responding cells were defined as those that exhibited a Δ*F*/*F*_max_ greater than 3.5 times the pre-stimulus root-mean-square noise level.

Two types of plots were generated for each 300-s experiment: a histogram showing the percentage of responding cells over time and a scatter plot indicating the time at which each cell first responded to the stimulus. Responding cells in these plots were arranged with respect to their distance from the transducer focus.

### Pharmacology

To investigate the mechanism of ultrasound-induced calcium rise in invasive cancer cells, PC-3 and T24/83 cells were stimulated in the presence of various pharmacological agents. We tested five different blockers, each applied separately (Table 1). Blockers were dissolved in the external buffer solution 15–30 min before performing imaging and ultrasound stimulation. Cellular responses were measured before adding the blockers, in the presence of blockers, and after washout.

**Table 1:** Pharmacological agents used to investigate the mechanism of ultrasound-induced calcium rise in invasive cancer cells.

Agent	Effect	Concentration (μM)	Loading time (min)	Reference
2-aminoethoxydiphenyl borate (2-APB)	Transient receptor potential channel blocker; IP_3_ receptor antagonist	100	15	(24, 25)
Cadmium chloride (CdCl_2_)	Voltage-gated Ca^2+^ channel blocker	20	15	(26)
Gadolinium (III) chloride (GdCl_3_)	Stretch-activated Ca^2+^ channel blocker	10	30	(27)
Iberiotoxin	BK_Ca_ channel blocker	0.1	15	(28)
Streptomycin	Stretch-activated Ca^2+^ channel blocker	200	30	(27)

## Results

### Matrigel Boyden Chamber Assays

**Figure 2:**
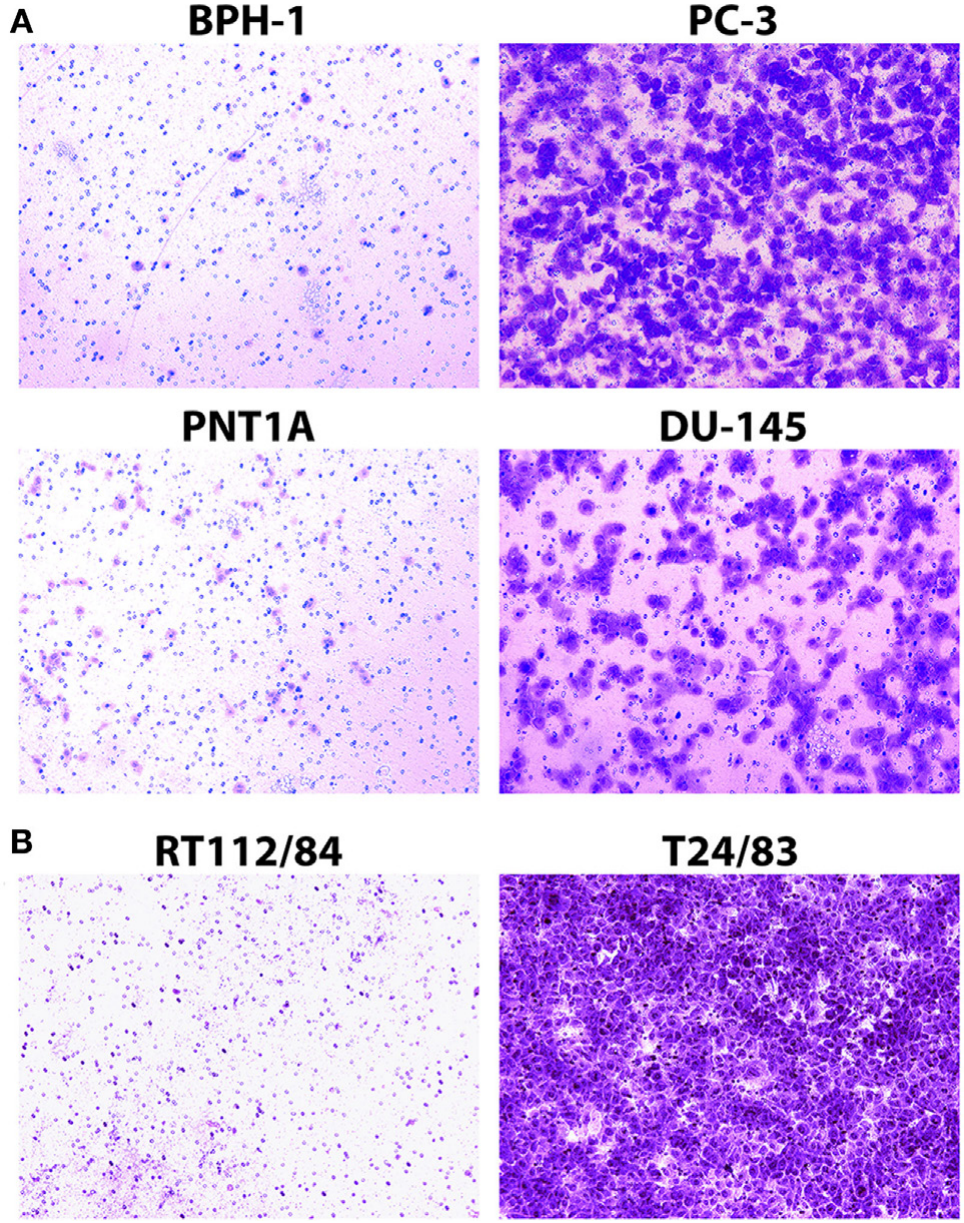
Assessment of prostate cancer **(A)** and bladder cancer **(B)** cell line invasion potential with Matrigel Boyden chamber assays. Cells that passed through the Matrigel barrier were stained with crystal violet. Results indicate that PC-3, DU-145, and T24/83 cells are highly invasive, while BPH-1, PNT1A, and RT112/84 cells are not. **(A)** Photomicrographs showing assay results for BPH-1, PNT1A, PC-3, and DU-145 prostate cancer cells. **(B)** Photomicrographs showing assay results for RT112/84 and T24/83 bladder cancer cells. One representative image for each cell line is shown.

The Matrigel Boyden chamber assay (20) is the standard technique for assessing cancer cell invasion potential in the laboratory setting. We used this assay to measure the invasion potential of four prostate cancer cell lines (PC-3, DU-145, BPH-1, and PNT1A) and two bladder cancer cell lines (T24/83 and RT112/84) with varying levels of invasiveness. As expected, PC-3, DU-145, and T24/83 cells exhibited strong Matrigel invasion, while BPH-1, PNT1A, and RT112/84 cells did not (Figure 2; also see Figure S1 in Supplementary Material) (16–19).

Despite the simplicity of the Matrigel Boyden chamber assay, it is not suitable for clinical applications. The assay requires at least 10–25 thousand cells, necessitating time-consuming establishment of cell cultures from tumor biopsies (29, 30), which is not always possible. Furthermore, the Matrigel assay is relatively slow, taking at least 24 h to complete. Our mechanotransduction assay overcomes these limitations, as it can be completed in a matter of minutes and does not require large numbers of cells.

### Calcium Responses to Ultrasound Stimulation Differ between Strongly and Weakly Invasive Prostate Cancer Cells

**Figure 3:**
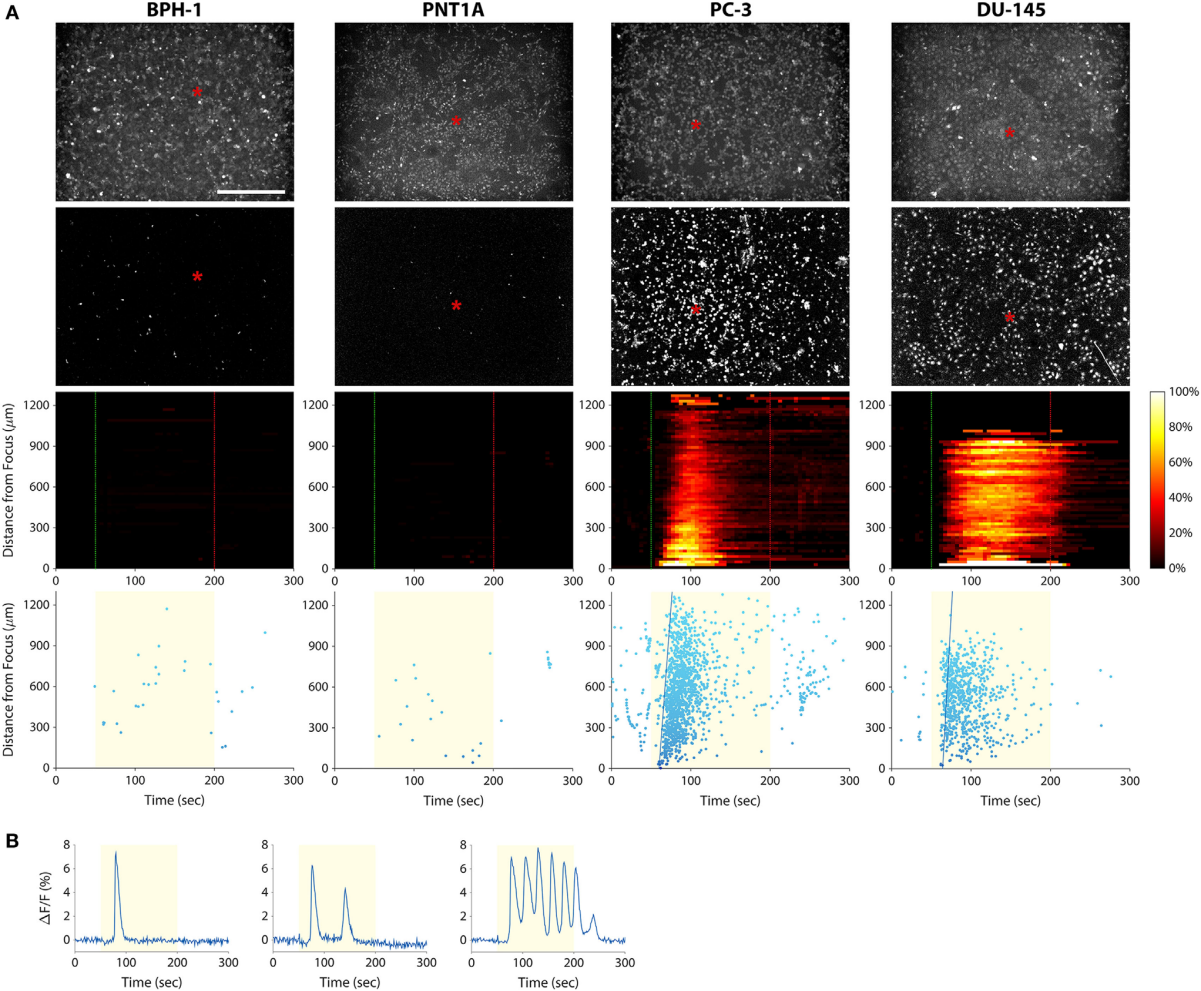
Measuring prostate cancer cell line invasion potential with our mechanotransduction assay. **(A)** BPH-1 (first column), PNT1A (second column), PC-3 (third column), and DU-145 (fourth column) cells were stimulated with 38-MHz low-intensity focused ultrasound while monitoring their activity with calcium imaging. Top row, baseline (pre-stimulation) fluorescence images of the cells. Red asterisks indicate the center position of the ultrasound focus. Scale bar is 500 µm. Second row, background-subtracted fluorescence images revealing all the cells that responded to the ultrasound stimulus (also see Videos S1–S4 in Supplementary Material). Only the PC-3 and DU-145 cells responded strongly, indicating that they are invasive. Third row, two-dimensional histograms showing the percentage of responding cells over time with respect to their distance from the transducer focus. Dotted green and red lines indicate stimulus onset and offset times, respectively. Bottom row, scatter plots showing the time at which each cell first responded to the stimulus (each dot represents a cell). The yellow shaded area indicates the times during which ultrasound was applied. Fitted blue lines indicate the speed of the calcium wave (77 µm/s in PC-3; 106 µm/s in DU-145). The histogram and scatter plots provide an intuitive means to visualize responses of the cell populations over time, making it easy to distinguish responding (invasive) cells from non-responding (non-invasive or weakly invasive) ones. **(B)** Fluorescence responses of three PC-3 cells showing the different types of calcium transients: single (left), double (middle), and oscillating (right).

To investigate the use of mechanotransduction for determining the invasion potential of tumor cell populations, we imaged calcium activity of PC-3, DU-145, BPH-1, and PNT1A prostate cancer cells during stimulation with 38-MHz low-intensity focused ultrasound. The stimulus consistently evoked strong calcium responses in highly invasive PC-3 and DU-145 cells, but not in weakly invasive BPH-1 and PNT1A cells (Figure 3; Videos S1–S4 in Supplementary Material). In most cases, PC-3 and DU-145 stimulation evoked an intercellular calcium wave that emanated from the transducer focus and spread at a speed of ~50–100 μm/s. The wave began 5–25 s after stimulation onset and persisted for 1–2.5 min. Despite the acoustic focus being localized to an area approximately 150 µm in diameter (see Figure 1B), the calcium wave propagated over distances greater than 1 mm. Most cells that responded to ultrasound stimulation exhibited a single calcium transient, though double transients and calcium oscillations were also observed (Figure 3B).

### Calcium Responses of Bladder Cancer Cell Lines Mirror Those of Prostate Cancer Cell Lines

**Figure 4:**
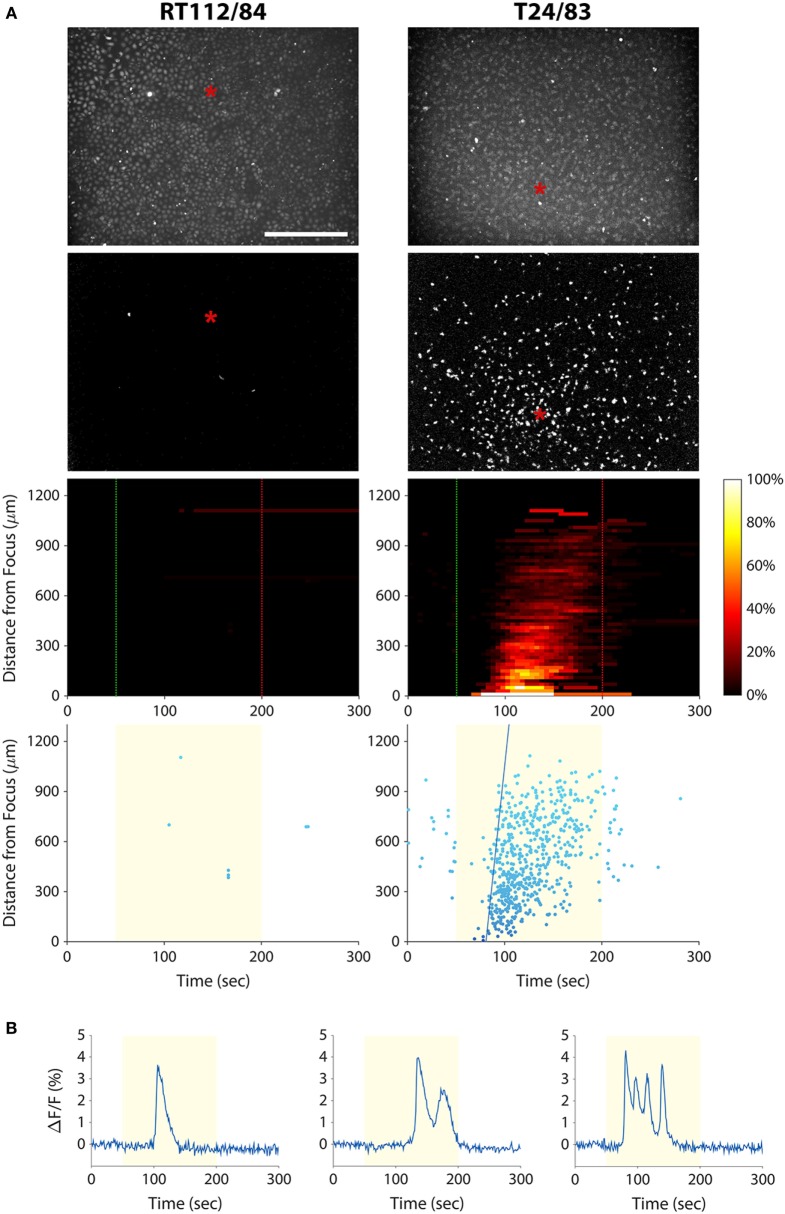
Measuring bladder cancer cell line invasion potential with our mechanotransduction assay. **(A)** RT112/84 (left column) and T24/83 (right column) cells were stimulated with 38-MHz low-intensity focused ultrasound while monitoring their activity with calcium imaging. Layout of the plots is the same as in Figure 3A (see Figure 3 caption for descriptions). Results indicate that T24/83 cells are highly invasive, while RT112/84 cells are not. The speed of the calcium wave induced in T24/83 cells was 55 µm/s. See also Videos S5 and S6 in Supplementary Material. **(B)** Fluorescence responses of three T24/83 cells showing the different types of calcium transients: single (left), double (middle), and oscillating (right).

As discussed above, a cytological assay of tumor cell invasion potential could have important implications for bladder cancer diagnosis and management. We therefore investigated whether the mechanotransduction assay could be applied to bladder cancer cells. We stimulated T24/83 (highly invasive) and RT112/84 (weakly invasive) bladder cancer cell lines with 38-MHz ultrasound while performing calcium imaging. As shown in Figure 4A and Videos S5 and S6 in Supplementary Material, the stimulus evoked a calcium wave in T24/83 cells, but no responses were observed in RT112/84 cells. The pattern and timing of T24/83 responses were similar to those of PC-3 prostate cancer cells (see Figure 3).

### Varying Stimulus Amplitude

**Figure 5:**
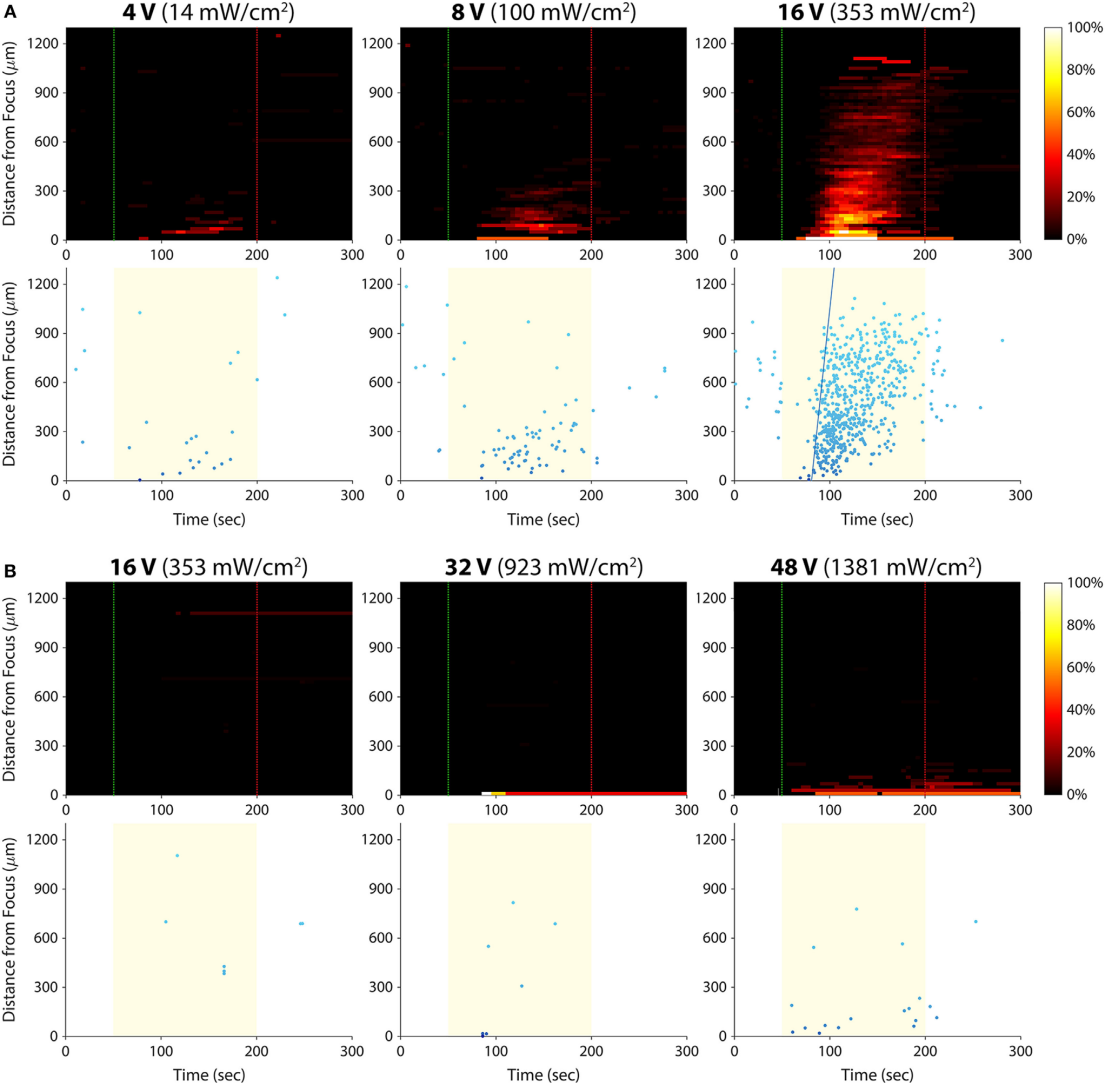
Effect of ultrasound stimulus amplitude on bladder cancer cell calcium responses. Standard stimulus parameters (see Materials and Methods) were used while varying the transducer input voltage. All stated voltages represent peak-to-peak amplitude. Values in parentheses indicate the *I*_spta_ at each voltage, as measured by a hydrophone. **(A)** Histogram and scatter plots from T24/83 cell stimulation. **(B)** Histogram and scatter plots from RT112/84 cell stimulation.

We hypothesized that there was an acoustic activation threshold (i.e., intensity) below which invasive cancer cells would not exhibit calcium responses to ultrasound stimulation. To test for such a threshold, we stimulated T24/83 cells at amplitudes lower than 16 V_p–p_ (the voltage used in all prior experiments; Figure 5A). The smallest amplitude we tested, 2 V_p–p_, did not evoke any detectable calcium activity. At 4 V_p–p_, the stimulus activated just a few cells in the area where the ultrasound was focused. Stimulation at 8 V_p–p_ also elicited responses near the focus, but more cells responded at this amplitude. Increasing the voltage to 16 V_p–p_ evoked hundreds of responses in the form of an intercellular calcium wave.

Given that RT112/84 bladder cancer cells are weakly invasive (19), we hypothesized that they might exhibit calcium responses at intensities greater than 16 V_p–p_. As shown in Figure 5B, stimulation at 16 V_p–p_ did not evoke any calcium activity in these cells (the few data points on the histogram and scatter plots are false positives arising from movement of hyperfluorescent debris in the Petri dish; see Video S5 in Supplementary Material). At 32 V_p–p_, three cells at the center of the transducer focus responded. At 48 V_p–p_, 10 –15 cells at the focus responded, but there was still no calcium wave. Amplitudes higher than 48 V_p–p_ were not tested, as they likely would have damaged the ultrasound transducer.

These results reveal a dose–response relationship between stimulus amplitude and the strength of the calcium responses. For a given invasion potential, there appears to be an acoustic activation threshold (inflection point of the dose–response curve) below which no or a few cells respond to ultrasound stimulation. Given the inverse relationship between acoustic activation threshold and invasion potential, it may be possible to quantify the invasion potential of a tumor cell population by measuring its acoustic activation threshold.

### Varying Stimulus Frequency

**Figure 6:**
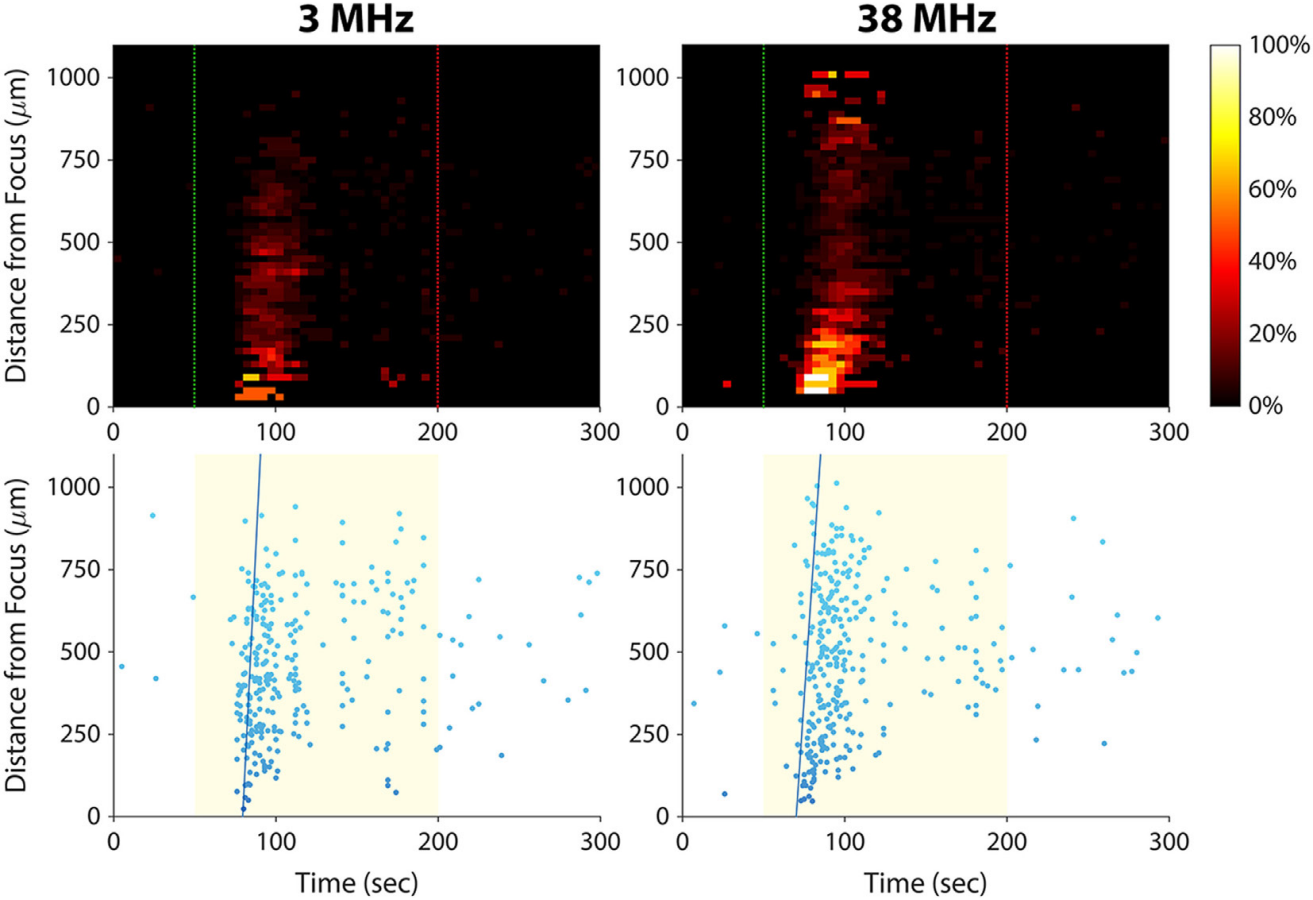
Effect of ultrasound stimulus frequency on prostate cancer cell calcium responses. The same set of PC-3 cells (same imaging field of view) was stimulated with different ultrasound transducers at frequencies of 3 MHz [96 V_p–p_ amplitude, 1 kHz pulse repetition frequency (PRF), 10% duty cycle] and 38 MHz (16 V_p–p_ amplitude, 1 kHz PRF, 5% duty cycle). The speed of the calcium wave induced at each frequency was 101 µm/s at 3 MHz and 73 µm/s at 38 MHz. Slightly fewer cells responded at 3 MHz than at 38 MHz (278 versus 311).

In this study, we have shown that 38-MHz focused ultrasound evokes calcium responses in invasive cancer cells, and we previously reported a similar effect for 200-MHz ultrasound (15). As shown in Figure 6, 3-MHz stimulation was also effective in eliciting calcium responses in invasive cells. Stimulating at 3 MHz evoked a calcium wave that propagated at 101 µm/s, similar to the speed of the calcium waves induced by 38-MHz stimulation (~50–100 μm/s; see Figures 3A and 4A). These results suggest that the mechanism of ultrasound-induced calcium rise in invasive cancer cells is at least partly independent of stimulus frequency.

### Mechanism of Ultrasound Stimulation

The fact that our assay can be applied to more than one cancer type (prostate and bladder) implies a conserved mechanism by which invasive cancer cells transduce ultrasound stimuli. To elucidate this mechanism, we applied pharmacological blockers of proteins we suspected were involved in the mechanotransduction process. We stimulated PC-3 and T24/83 cells in the presence of five different blockers, each applied separately (Table 1). We blocked voltage-gated Ca^2+^ channels, which are known to be mechanosensitive (31), as well as stretch-activated Ca^2+^ channels. We also blocked BK_Ca_ channels, which are stretch activatable (32) and expressed in PC-3 cells (33). Finally, we applied a drug that blocks both transient receptor potential (TRP) channels and inositol trisphosphate (IP_3_) receptors. TRP channels are mechanosensitive, permeable to calcium, and exhibit altered expression levels in several types of cancer (13, 34, 35). IP_3_ receptors are found on the endoplasmic reticulum and mediate Ca^2+^ release from intracellular stores.

**Figure 7:**
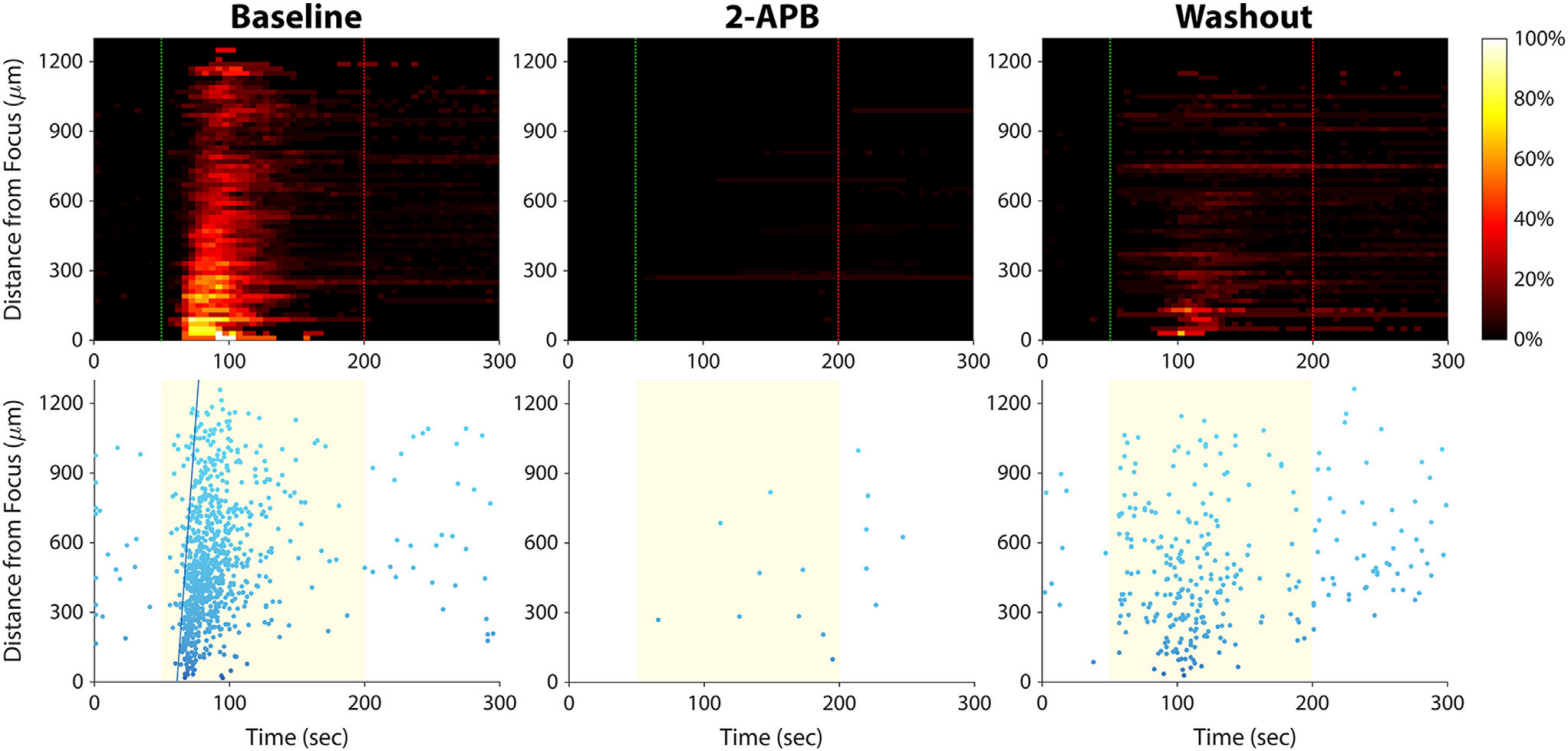
Effect of 2-APB on ultrasound-stimulated calcium responses in PC-3 prostate cancer cells. Left column, the cells exhibited strong calcium responses at baseline, before the drug was applied. Center column, cells were stimulated again 15 min after 2-APB (100 µM) application, and no responses were observed (the few data points on the histogram and scatter plots are false positives arising from movement of hyperfluorescent debris in the Petri dish). Right column, 30 min after 2-APB washout, ultrasound-stimulated calcium responses were partially restored. Results in T24/83 bladder cancer cells were similar.

Of the five blockers we tested (Table 1), only 2-aminoethoxydiphenyl borate (2-APB) had an effect on the calcium responses. Application of 2-APB abolished all ultrasound-induced calcium activity, an effect that was partially reversed upon washout of the drug (Figure 7). This indicates that TRP channels and/or IP_3_ receptors are involved in mediating invasive cancer cell responses to ultrasound stimulation (see Discussion).

### Mechanism of the Calcium Waves

Intercellular calcium waves are common biological phenomena that occur in many cell types. They play a role in a variety of cellular activities including migration and mechanotransduction (36). Calcium waves typically occur *via* transmission of an intracellular messenger such as IP_3_ through gap junctions (37), or by release of an extracellular messenger such as adenosine triphosphate that diffuses to surrounding cells (38). In the case of our experiments, there was a third possibility—that ultrasound energy impinging upon the Petri dish surface was being reflected by the substrate and generating a surface wave that was activating distant cells.

**Figure 8:**
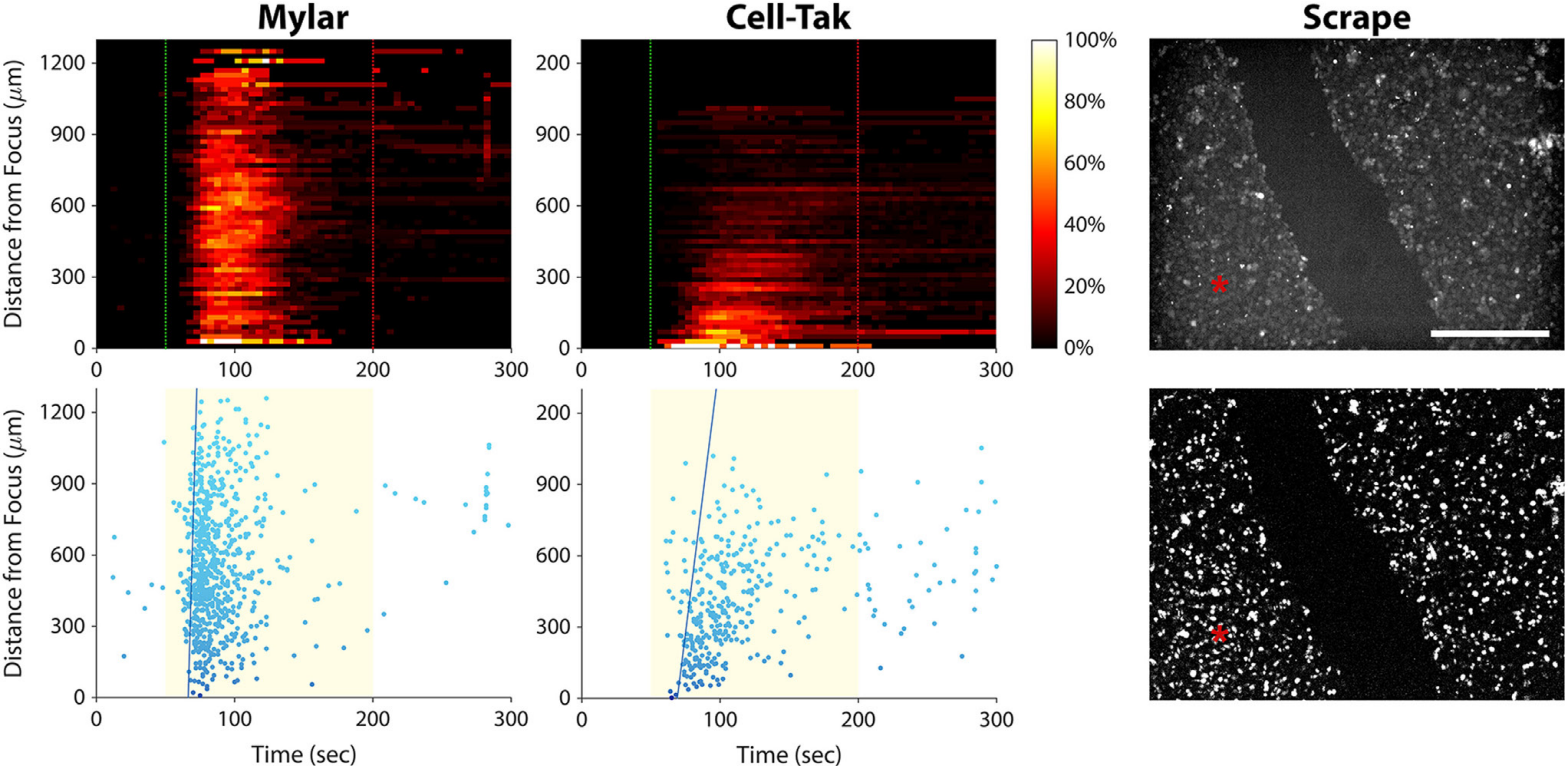
Elucidating the cause of the calcium wave in PC-3 cells. Left column, histogram, and scatter plots showing a calcium wave in cells seeded on a Mylar substrate. This result indicates that the wave is not caused by ultrasound reflection by the substrate. Center column, histogram, and scatter plots showing ultrasound responses of cells freshly seeded on a Cell-Tak-coated Petri dish. A calcium wave occurs even in the absence of gap junctions. Right column, baseline (top), and background-subtracted (bottom) fluorescence images of ultrasound responses occurring after scraping a channel of cells off the substrate. Red asterisks indicate the center position of the ultrasound focus. Calcium responses occurred on both sides of the gap, suggesting that calcium waves in invasive cancer cells are caused by release of signaling molecules that diffuse through the extracellular space. Scale bar is 500 µm.

To rule out the possibility of ultrasonic surface wave induced activation, we stimulated PC-3 cells seeded on an acoustically transparent Mylar film, thus minimizing ultrasound reflection by the substrate. As shown in Figure 8 (left column), the ultrasound stimulus still evoked a calcium wave in these Mylar-seeded cells. This indicates that the wave was likely caused by cell-to-cell signaling, either through gap junctions or *via* release of an extracellular messenger.

To determine whether the calcium wave was propagating through gap junctions, we seeded PC-3 cells on a Petri dish coated with Cell-Tak Cell and Tissue Adhesive, in order to facilitate immediate cell adhesion. We then stimulated the cells within minutes, before they could form gap junctions. The calcium wave was not eliminated under these circumstances (Figure 8, center column), ruling out the possibility that it was propagating through gap junctions.

To test whether extracellular signaling molecules were generating the calcium wave, we used a pipette tip to scrape a channel of PC-3 cells off the substrate, leaving two regions of confluent cells separated by a 400-µm gap (Figure 8, top right). We then stimulated the cells on one side of the gap with ultrasound. The stimulus evoked a calcium wave that propagated across the gap, eliciting responses in cells on the other side (Figure 8, bottom right). These results support the notion that the calcium waves observed in invasive cancer cells are mediated by signaling molecules that diffuse through the extracellular solution.

## Discussion

We have demonstrated a new method for assessing the invasion potential of cancer cell populations and have validated the approach in prostate and bladder cancer cell lines. To our knowledge, this is the first technique other than the Matrigel Boyden chamber assay that can assess invasion of isolated tumor cells (as opposed to intact tissue). Unlike the Matrigel assay, our assay is rapid and does not require tens of thousands of cells. It can measure invasion potential of single cells as we have shown previously (15), or as shown in the present study, it can be applied to cancer cell populations.

We are not the first to investigate how low-intensity ultrasound interacts with invasive cancer cells. Tran et al. stimulated invasive MDA-MB-231 breast cancer cells with 1-MHz ultrasound (up to 500 kPa) while using the patch clamp technique to monitor membrane potential (32, 39). The cells immediately became hyperpolarized upon ultrasound application, but only when they were in direct contact with gas-filled microbubbles. By applying the BK_Ca_ channel blocker iberiotoxin, the authors determined that the microbubbles were activating mechanically sensitive BK_Ca_ channels, causing K^+^ efflux and consequent hyperpolarization (32).

The BK_Ca_-based mechanotransduction mechanism reported by the Tran study is fundamentally different from the mechanism observed in our study: We observed ultrasound responses in invasive cancer cells without using microbubbles. Furthermore, we found that PC-3 and T24/83 responses were not affected by iberiotoxin (Table 1), thus excluding involvement of BK_Ca_ channels in the mechanotransduction process. Instead, we found that the drug 2-APB blocked ultrasound-induced calcium activity (Figure 7), implicating a role of TRP channels and/or IP_3_ receptors. We also observed a delay of several seconds between stimulus onset and the calcium responses, suggesting a second messenger effect.

Several lines of evidence support the possibility that TRP channels are mediating the ultrasound responses observed in our study. TRP channels are a family of non-selective cation channels that can be activated by mechanical force, either directly or through a second messenger pathway (34). They have been implicated in several types of cancer including prostate (40) and bladder (41) cancer, regulating behaviors such as proliferation, differentiation, and migration [see Ref. (13, 35) for review]. Invasive and metastatic cancers are known to overexpress certain TRP channel isoforms, and silencing expression of these isoforms in cell lines significantly reduces migration and invasion (42, 43). It was recently reported that TRP-4, the *C. elegans* TRP channel homolog, transduces low-pressure (≤900 kPa) ultrasound stimuli in this model organism [though only in the presence of microbubbles; Ref. (44)].

Because 2-APB blocks both TRP channels and IP_3_ receptors, it is possible, however, that IP_3_ receptors are involved in transducing the ultrasound stimulus (instead of or in addition to TRP channels). IP_3_ and its receptors play a dominant role in transducing external stimuli into calcium signals by evoking Ca^2+^ release from intracellular stores. They are also known to mediate oscillatory changes in cytosolic calcium concentration (45), such as those observed in this study (see Figures 3B and 4B). In ongoing work, we aim to determine the precise mechanism of ultrasound-induced calcium rise *via* small interfering RNA-mediated downregulation of IP_3_ receptors versus TRP channels.

Although we found that ultrasound stimulation consistently evoked calcium waves in invasive cancer cells, the timing of those waves was somewhat variable. Calcium waves typically began 5–25 s after stimulation onset, traveled at a speed of ~50–100 μm/s, and persisted for 1–2.5 min. Though the reason for this variability is not clear, it is possible that the calcium wave timing correlates with invasion potential (for example, a faster wave could indicate greater invasion potential). In support of this theory, we did observe spontaneous variations in the degree of PC-3 and T24/83 Matrigel invasiveness that occurred during passaging in culture (data not shown). Likewise, others have reported that prostate and bladder cancer cells undergo cyclical, population-wide changes in tumorigenicity as they are passaged (46). Future studies will explore whether these changes in tumorigenicity and Matrigel invasiveness correlate with the pattern of ultrasound responses (such as calcium wave speed, percentage of responding cells, etc.).

If validated using clinical specimens, our mechanotransduction assay could provide pathologists with a means to detect tumor invasion in cytology specimens, a feat that is not currently possible. At present, pathologists can assess invasion only through histological analysis of biopsied tissue. However, there are many instances when intact tissue cannot be obtained from the patient. In such cases, diagnosis often relies exclusively on cytology, which cannot assess invasion. This can have devastating consequences, for example in the case of recurrent bladder cancer. As discussed above, recurrent invasive bladder malignancies sometimes go undetected by cystoscopy, which can be life-threatening (1, 4). An assay for identifying invasion in bladder wash cytology specimens could inform treatment decisions that improve patient outcomes. Esophageal carcinoma is another disease that could benefit from cytological assessment of tumor invasion. Detecting invasion in cells collected from esophageal brushings could limit the need for endoscopic tumor biopsies, which carry the serious risk of esophageal perforation (47).

In addition to assessing invasion potential, our technology could also be as a high-throughput optical screen for drugs that target the mechanotransduction pathway (48). Cells could be placed in multiwell plates, treated with different drugs, and then stimulated with ultrasound while imaging calcium activity. Drug effects would be indicated by any changes induced in the pattern of ultrasound responses.

A functional assay of cancer cell invasion potential, as demonstrated herein, could provide key advantages over other types of cancer diagnostic assays. Genomic tests, for example, are intended to predict tumor aggression or recurrence and have gained widespread use in recent years. These tests are limited, however, in that they provide information about nucleic acid expression, rather than protein expression/translation or protein functional state, which can be altered post-translationally. Protein activity is what controls a tumor’s behavior, including its ability to invade and metastasize (5). By probing cell function as a measure of invasion potential, our assay may provide a more precise measure of a tumor’s propensity to spread.

It may eventually be possible to develop an *in vivo* version of the assay that could mitigate the need for tumor biopsies. In the case of bladder cancer, a confocal laser endoscope (49) with an integrated ultrasound transducer could deliver calcium dye to the tumor, stimulate it with ultrasound, and image cellular responses. For other types of solid cancers, the tumor could be stimulated percutaneously with ultrasound while using calcium-sensitive MRI contrast agents (50) to image the response. This approach would provide an entirely non-invasive means to assess tumor invasion potential.

## Author Contributions

AW, NL, CY, AB, KG, SM, and HJ collected and analyzed data. SK analyzed data. AW, NL, CY, QZ, RC, and KS designed the research. AW and NL wrote the manuscript. All authors read, contributed to, and approved the final manuscript.

## Conflict of Interest Statement

AW, NL, RC, and KS have applied for a patent (US 14/040,253) related to this work.

## Supplementary Material

Figure S1 Matrigel Boyden chamber assays of BPH-1 cells at different time points. Cells that passed through the Matrigel barrier were stained with crystal violet. As cells were passaged in culture, their level of invasiveness changed spontaneously over time. To obtain a weakly invasive homogeneous population for use in ultrasound stimulation experiments, BPH-1 cells that did not pass through the Boyden chamber were selected and propagated (see Materials and Methods).

Video S1 Calcium responses of weakly invasive BPH-1 prostate cancer cells to stimulation with 38-MHz low-intensity focused ultrasound (video corresponds to images in Figure 3, first column). The red asterisk indicates the center position of the ultrasound focus; its appearance and disappearance coincide with stimulation onset and offset, respectively. Video is played back at 60× real-time speed.

Video S2 Calcium responses of weakly invasive PNT1A prostate cancer cells to stimulation with 38-MHz low-intensity focused ultrasound (video corresponds to images in Figure 3, second column). The red asterisk indicates the center position of the ultrasound focus; its appearance and disappearance coincide with stimulation onset and offset, respectively. Video is played back at 60× real-time speed.

Video S3 Calcium responses of strongly invasive PC-3 prostate cancer cells to stimulation with 38-MHz low-intensity focused ultrasound (video corresponds to images in Figure 3, third column). The red asterisk indicates the center position of the ultrasound focus; its appearance and disappearance coincide with stimulation onset and offset, respectively. Video is played back at 60× real-time speed.

Video S4 Calcium responses of strongly invasive DU-145 prostate cancer cells to stimulation with 38-MHz low-intensity focused ultrasound (video corresponds to images in Figure 3, fourth column). The red asterisk indicates the center position of the ultrasound focus; its appearance and disappearance coincide with stimulation onset and offset, respectively. Video is played back at 60× real-time speed.

Video S5 Calcium responses of weakly invasive RT112/84 bladder cancer cells to stimulation with 38-MHz low-intensity focused ultrasound (video corresponds to images in Figure 4, left column). The red asterisk indicates the center position of the ultrasound focus; its appearance and disappearance coincide with stimulation onset and offset, respectively. Video is played back at 60× real-time speed.

Video S6 Calcium responses of strongly invasive T24/83 bladder cancer cells to stimulation with 38-MHz low-intensity focused ultrasound (video corresponds to images in Figure 4, right column). The red asterisk indicates the center position of the ultrasound focus; its appearance and disappearance coincide with stimulation onset and offset, respectively. Video is played back at 60× real-time speed.
