# Supplementary figures and images for: Functional Assay of Cancer Cell Invasion Potential Based on Mechanotransduction of Focused Ultrasound
Source: Front Oncol. 2017 Aug 7;7:161. doi: 10.3389/fonc.2017.00161 (PMC5545605; doi:10.3389/fonc.2017.00161)

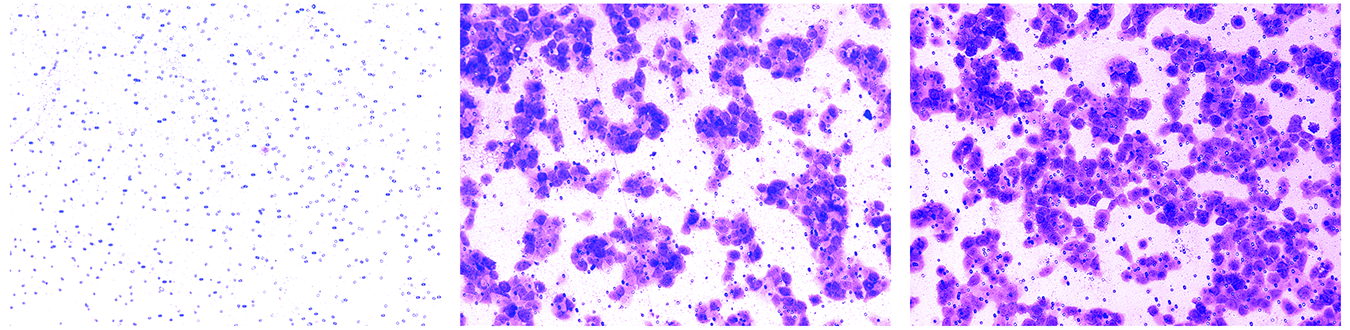

Supplement: Figure S1 — Matrigel Boyden chamber assays of BPH-1 cells at different time points. Cells that passed through the Matrigel barrier were stained with crystal violet. As cells were passaged in culture, their level of invasiveness changed spontaneously over time. To obtain a weakly invasive homogeneous population for use in ultrasound stimulation experiments, BPH-1 cells that did not pass through the Boyden chamber were selected and propagated (see Materials and Methods). [file image_1.tif]
